# Determination of the appropriate propofol infusion rate for outpatient upper gastrointestinal endoscopy-a randomized prospective study

**DOI:** 10.1186/s12876-016-0463-y

**Published:** 2016-04-26

**Authors:** Qiongzhen Li, Qinghe Zhou, Wangpin Xiao, Hongmei Zhou

**Affiliations:** Department of Anesthesia, Shanghai Chest Hospital, Shanghai Jiao Tong University, Shanghai, China; Department of Anesthesia, Second Hospital, Jiaxing Medical College, No. 1518, North Huancheng Road, Jianshe Street, Nanhu District, Jiaxing, 314000 Zhejiang China

**Keywords:** Propofol, Outpatient, Upper gastrointestinal endoscopy, Sedation, Complications

## Abstract

**Background:**

Pain and discomfort related to endoscopy sessions can be alleviated by sedation, which minimizes anxiety and allows safe examination. For outpatient endoscopy, reliable short-term sedation without secondary effects is required. This study aimed to assess the effects of intravenous propofol rates on sedation in outpatients undergoing upper gastrointestinal endoscopy.

**Methods:**

This randomized prospective study evaluated 300 outpatients submitted to upper gastrointestinal endoscopy. Patients received propofol at 500, 1000 or 2000 ml/h. The primary outcome assessed was hypoxemia incidence. In addition, time to sedation and incidence of hypotension, deep sedation, extremity motor activity, cough, nausea, hiccough, and awareness were evaluated.

**Results:**

Recovery time and incidence of hypoxemia, hypotension, and deep sedation were significantly increased in individuals treated at 2000 ml/h in comparison with values obtained for 500 and 1000 ml/h groups (*P* < 0.01). Compared with the 500 ml/h group, motor activity of the extremities, cough, nausea, hiccough, and awareness were significantly decreased and the mean scores for endoscopist’s and patients’ satisfaction were significantly increased in the 1000 and 2000 ml/h groups (*P* < 0.01).

**Conclusion:**

Propofol infused at 1000 ml/h appeared to be the most suitable infusion rate for outpatient upper gastrointestinal endoscopy.

**Trial registration:**

Registration number: ChiCTR-TRC-14004786; Registration date: 2014-06-04

## Background

Pain and discomfort related to endoscopy sessions can be alleviated by sedation, which minimizes anxiety and allows safe examination [1]. Over 98 % of endoscopies are performed under sedation [2, 3]. For outpatient endoscopic procedures, reliable short term sedation without secondary effects is required. Intravenous (IV) propofol is frequently used [4, 5].

Propofol (2, 6-diisopropylphenol) is a hypnotic agent that induces anesthesia in about 50 s at standard doses [6]. Propofol is characterized by short half-life, rapid onset, important volume of distribution, and absence of active metabolites, leading to rapid recovery and discharge. These properties make propofol suitable for outpatient gastrointestinal endoscopy.

Using the same dose, the final blood concentrations of propofol depend on patient sex, age and weight, and drug dose, administration rate, and cardiac output [7–10]. Previous studies have shown that the required doses for anesthesia onset are affected by changes in the rate of injection [7, 10, 11]. A previous study has shown that the hypnotic peak effect of propofol is lower using a very slow injection rate (240 s), but there was no difference when using clinically-used injection rates (5–120 s) [12]. Another study showed that the sleep dose of propofol is reduced using slower infusion rates, while fast infusion rates resulted in greater decreases in heart rate and higher incidence of apnea [13]. The use of a slow infusion rate results in longer induction duration, but requires a lower total dose, and results in a lower incidence of apnea and a lesser decrease in blood pressure [14]. However, the appropriate injection rate in an outpatient setting is still controversial.

This study aimed to identify the most suitable propofol infusion rate for outpatients submitted to upper gastrointestinal endoscopy that resulted in the lowest adverse effect incidence and was associated with the highest patient satisfaction.

## Methods

### Study design

This randomized, parallel, controlled study was performed at the Department of Anesthesia, Second Hospital, Jiaxing Medical College (Jiaxing, Zhejiang, China) between June 2014 and August 2014 (registration number ChiCTR-TRC-14004786). The procedures were approved by the ethics review board of the Second Affiliated Hospital of Jiaxing College, chaired by Professor Liqin Jiang (number CZJ 65). Written informed consent was obtained from each patient prior to participation.

### Patients

For patient inclusion, the following criteria were set: 1) American Society of Anesthesiologists (ASA) class I-III; 2) upper gastrointestinal endoscopy; and 3) age 18–65 years. Exclusion criteria were: 1) pregnancy; 2) allergies to eggs, beans, or latex; 3) history of alcohol or sedative overdose [15]; 4) history of adverse events associated with propofol; 5) sleep apnea or acute gastrointestinal hemorrhage; or 6) recent abnormalities of the central nervous system such as stroke.

### Intervention

The patients were randomized to one of three groups using a random number table. Patients were administered oxygen at a rate of 2–3 L/min using a nasal catheter before induction. Propofol (Xi’an Libang Shaanxi, China; batch number: 1405282) was administered as an IV bolus by a sub-senior anesthesiologist (13 years of experience) at 500 (500 ml/h group), 1000 (1000 ml/h group) or 2000 ml/h (2000 ml/h group) to unconsciousness. Upon loss of consciousness, rapid infusion was stopped. If the patient still had body movements after infusion, an additional 20–30 mg of propofol was administered to avoid body movements during the procedure. The same senior endoscopist (18 years of experience) performed all procedures with the help of the same supervisor nurse (15 years of experience). The endoscopist had no access to grouping information. The anesthesiologist that administered the additional propofol was blinded to patient’s history and initial induction infusion rate.

The target depth of sedation was loss of consciousness, no response to call, and eyelashes reflection. At this moment, propofol was stopped and the endoscope was inserted. If the patients had a body response upon endoscope insertion, propofol was added. The depth of sedation was evaluated according to body response and bispectral index (BIS) values.

### Monitoring

An arm vein of the patient was intubated with a 22G infusion catheter, and non-invasive monitoring of the arterial pressure was performed with the contralateral arm. Heart, respiratory, and oxygen saturation (SpO_2_) rates were continuously monitored. Induction dose corresponded to the drug amount delivered, and the rate was recorded (Smiths WZ-50F6 two-way injection pump; Zhejiang Smith Medical Instruments Co., Ltd., Ningxia, China). Any excitatory effects or periods of apnea (>30 s) after induction were recorded. Recovery time reflected the period from endoscopy end to full orientation (the patient was able to provide his/her correct date of birth).

The level of sedation was evaluated using BIS monitoring [16–18]. After skin disinfection, disposable electrodes were placed on the forehead, with the leads connected to a BIS Aspect monitor (Medical System, USA), whose output was assessed throughout the operation and recovery. BIS ≤65, 66–85, and >85 indicated deep, conscious, and mild sedation states, respectively. BIS levels were recorded at the following times: baseline, loss of consciousness, endoscope reaching the glottis, endoscope reaching the duodenum, end of endoscopy, and recovery. An independent observer (resident doctor, 3 years of experience, blind to grouping) was responsible for monitoring the patients including level of consciousness and adverse reactions (hypoxemia, bradycardia, hypotension, body movements, cough, nausea, hiccough, awareness) and for data collection (drugs, doses, and onset of cardiorespiratory events).

Adverse events were: hypoxemia (hypoxemia was defined SpO2 falling below 90 %), hypotension (systolic or diastolic blood pressure values decreasing by 20 % or more) and bradycardia (heart rate less than 50 bpm). Severe hypoxemia was defined as SpO2 declining below 90 %, while mild hypoxemia was defined as SpO2 declining below 90 % but rising to above 90 % after more than 30 s of jaw thrust maneuver.

As described by Cohen [19], we compared the three groups with respect to the time to induction (time period from first drug bolus dose to procedure initiation) and the time to recovery (time elapsed from endoscope removal to final evaluation). A patient was discharged only when BIS levels exceeded 90 and when there was no complaint of pain or discomfort.

At hospital discharge, a 10-point visual analogue scale (VAS) was used to evaluate patient satisfaction, from 1 to 10, representing least and most satisfied, respectively. Additionally, a VAS was used by the endoscopist to record his level of satisfaction with the sedation regimen. Respondents had to circle the number that corresponded the best to their experience. The endoscopist’s VAS was scored as 1–3, 4–7, and 8–10 for considerable, minor, and no difficulty in performing the procedure; respectively.

### Data collection

Age, sex, weight, height, ASA physical status, complications and adverse reactions during endoscopy, patient VAS score, and endoscopist VAS score were recorded. The following parameters were recorded during endoscopy: time to unconsciousness, total propofol dose, propofol dose (mg.kg^−1^), duration of endoscopy, recovery time, and BIS value at baseline, loss of consciousness, endoscope reaching the glottis, endoscope reaching the duodenum, end of endoscopy and recovery.

### Endpoints

The primary endpoint was the incidence of hypoxemia. The secondary endpoints were: incidence of adverse events other than hypoxemia (bradycardia, hypotension, body movement, cough, nausea, hiccough and, awareness); satisfaction of the endoscopist with sedation; BIS values; time to loss of consciousness; total propofol dose; pain during injection; endoscopy duration; and recovery time.

### Statistical analysis

Based on our preliminary experiments, the mean frequency of hypoxemia (primary outcome) was approximately 12 % during gastrointestinal endoscopy, and the sample size was calculated based on reducing this frequency by 50 %. To achieve 90 % power at an α level of 0.05 to detect a two-tailed difference, at least 88 patients were required in each group.

Data were analyzed using SPSS 19.0 (IBM, Armonk, NY, USA). Continuous data are mean ± standard deviation (SD). Categorical data were reported as frequencies. One-way analysis of variance (ANOVA) was employed for group comparisons, with the post hoc Bonferroni multiple comparison test. Categorical variables were assessed by the Pearson’s chi-square test. Two-tailed *P*-value ≤0.05 was considered statistically significant. Statistical review of this study was performed by a biomedical statistician (Tao Zhu).

## Results

### Characteristics of the patients

**Fig. 1:**
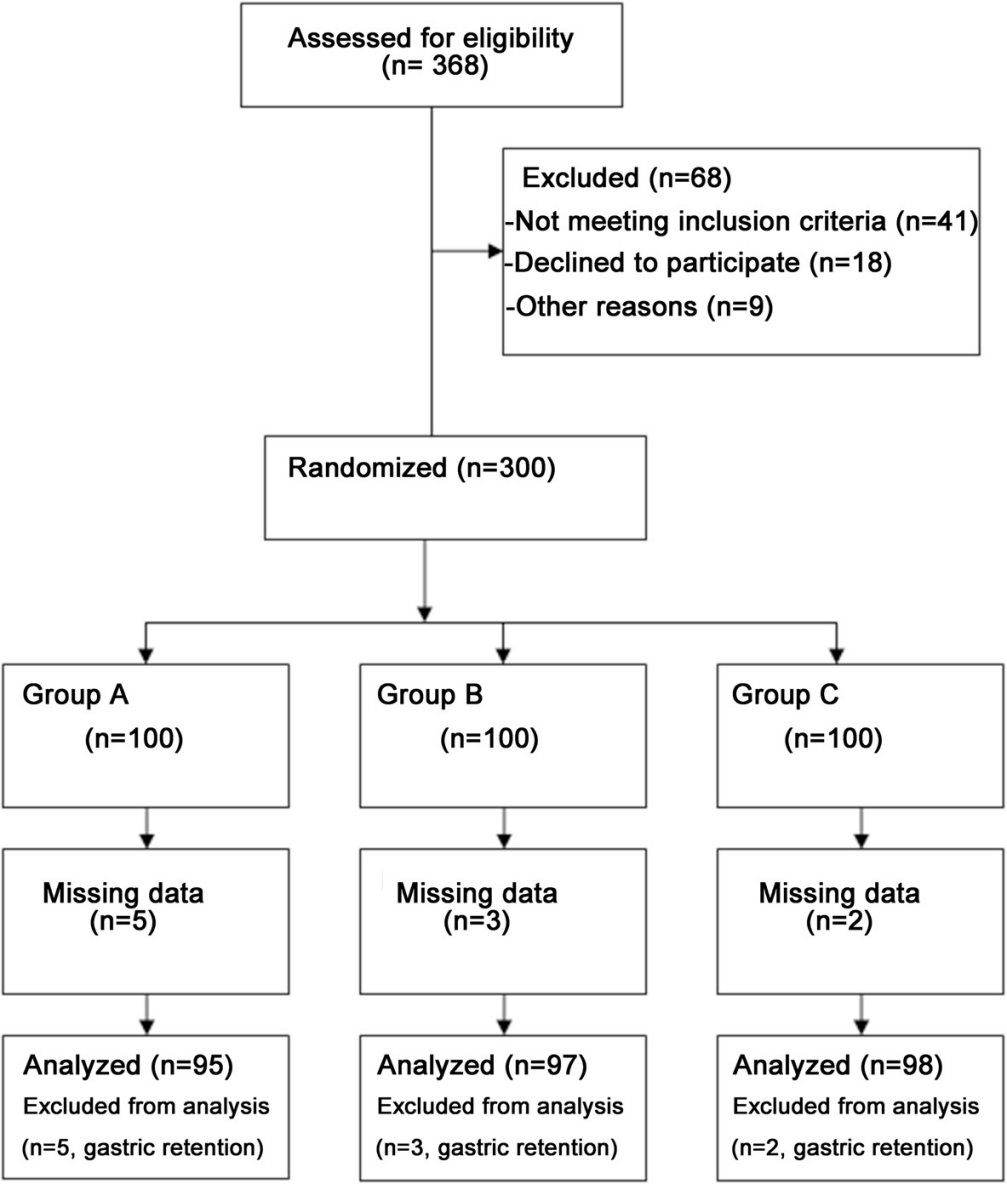
Patient flowchart

A total of 368 individuals were enrolled, with 41 not meeting the inclusion criteria; 18 withdrew from the study and 9 were excluded for unknown reasons. All patients underwent endoscopy for diagnostic procedures. The remaining 300 patients were randomized into one of the three groups (Fig. 1). Table 1 presents the characteristics of the patients. Patients were aged 50.1 ± 13.1, 52.0 ± 11.3 and 51.0 ± 11.3 years in the 500, 1000 and 2000 ml/h groups, respectively, and the proportion of females was 68.4, 72.2 and 64.3 %, respectively (all *P* > 0.05). There was no difference in weight, height, ASA class, body mass index (BMI), and endoscopy history (all *P* > 0.05).

**Table 1:** Characteristics of the patients

Variables	500 ml/h (*n* = 95)	1000 ml/h (*n* = 97)	2000 ml/h (*n* = 98)	*P*-value
Age (years)	50.1 ± 13.1	52 ± 11.3	51 ± 11.3	>0.05
Sex (M/F)	65/30	70/27	63/35	>0.05
Weight (kg)	63.6 ± 11.7	62.5 ± 8.1	61.9 ± 8.8	>0.05
Height (cm)	162.7 ± 7.1	161.9 ± 6.6	161.6 ± 6.7	>0.05
ASA class I/II/III	60/30/5	63/26/8	58/34/6	>0.05
BMI <16 or BMI >30 (kg m^−2^)	2/5	1/2	3/3	>0.05
Endoscopy history	65	72	70	>0.05

Data are expressed as means ± SD or number of patients. *ASA* American Society of Anesthesiologists

### Time to loss of conscious, dose, duration of endoscopy, and recovery time

Time to loss of consciousness was significantly longer with the slower infusion rates (86.4 ± 6.0 vs. 54.1 ± 4.8 vs. 32.4 ± 5.2 s at 500, 1000 and 2000 ml/h, respectively; all *P* < 0.01). Total (induction dose + eventual additional doses) and weight-related induction doses were significantly less with 500 and 1000 ml/h compared with 2000 ml/h (2.4 ± 0.4 and 2.4 ± 0.3 vs. 2.9 ± 0.5 mg/kg, *P* < 0.01). In comparison with patients treated at 500 ml/h, the duration of endoscopy was decreased in the 1000 and 2000 ml/h groups (*P* < 0.05). However, in comparison with individuals treated with propofol at 2000 ml/h, recovery times were shorter in the 500 and 1000 ml/h groups (18.1 ± 2.0 vs. 13.2 ± 1.6 and 13.1 ± 1.6 min, *P* < 0.05) (Table 2).

**Table 2:** Time to loss of consciousness, dosage, duration of endoscopy, and recovery time

Variables	500 ml/h	1000 ml/h	2000 ml/h
Time to loss of consciousness	86.4 ± 6.0	54.1 ± 4.8*	32.4 ± 5.2*^,^ **
Total propofol dose (induction dose + additional doses) (mg)	155.1 ± 15.4	150.8 ± 12.1	180.1 ± 19.5*^,^ **
Propofol dose (mg.kg^−1^)	2.4 ± 0.4	2.4 ± 0.3	2.9 ± 0.5*^,^ **
Duration of endoscopy (min)	4.0 ± 0.6	3.4 ± 0.6*	3.5 ± 0.6*
Recovery times (min)	13.2 ± 1.6	13.1 ± 1.6	18.1 ± 2.0*^,^ **

Data are expressed as means ± SD

*P < 0.01 vs. 500 ml/h

**P < 0.01 vs. 1000 ml/h

### Bispectral index values

Compared with the 500 and 1000 ml/h groups, BIS values were significantly decreased in patients receiving propofol at 2000 ml/h at loss of consciousness, endoscope reaching the glottis, endoscope reaching the duodenum and end of endoscopy (*P* < 0.01) (Table 3).

**Table 3:** Bispectral index value at different time points

Time points	500 ml/h	1000 ml/h	2000 ml/h
Baseline	96.0 ± 1.7	96.5 ± 1.5	96.1 ± 1.8
Loss of consciousness	80.0 ± 3.8	79.1 ± 3.7	75.9 ± 4.0*^,^ **
Endoscope reaching the glottis	75.8 ± 3.9	74.9 ± 3.5	69.0 ± 2.7*^,^ **
Endoscope reaching the duodenum	67.0 ± 2.9	68.0 ± 3.0	62.9 ± 4.5*^,^ **
End of endoscopy	71.2 ± 5.6	72.0 ± 5.4	67.8 ± 3.1*^,^ **
Recovery	87.6 ± 1.5	87.8 ± 1.5	87.5 ± 1.5

Data are expressed as means ± SD

*P < 0.01 vs. 500 ml/h

**P < 0.01 vs. 1000 ml/h

### Complications and adverse reactions

In comparison with the 2000 ml/h group, the frequencies of hypotension and hypoxemia were significantly decreased in patients administered propofol at 500 and 1000 ml/h (*P* < 0.01). However, no statistically significant differences were obtained between groups regarding arrhythmia and pain during injection (Tables 4 and 5). Compared with the 1000 and 2000 ml/h groups, the frequencies of extremity motor activity, cough, nausea, hiccough and awareness were significantly increased in patients treated at 500 ml/h (*P* < 0.01) (Table 4). Deep sedation rate was significantly increased after propofol administration at 2000 ml/h compared with the 500 and 1000 ml/h groups (*P* < 0.01) (Table 4).

**Table 4:** Complications and adverse reactions during endoscopy

Complications and adverse reactions	500 ml/h	1000 ml/h	2000 ml/h
Hypoxemia	10 (10.5)	15 (15.4)	40 (40.8)*^,^ **
Arrhythmia	0 (0.0)	1 (1.0)	3 (3.1)
Systolic hypotension	10 (10.5)	14 (14.4)	30 (30.6)*^,^ **
Diastolic hypotension	16 (16.8)	19 (19.6)	45 (45.9)*^,^ **
Extremity motor activity	33 (34.7)	9 (9.3)*	7 (7.1)*
Cough	16 (16.8)	5 (5.2)*	2 (2.0)*
Nausea	19 (20.0)	7 (7.2)*	4 (4.1)*
Hiccough	15 (15.8)	4 (4.1)*	3 (3.1)*
Awareness	17 (17.9)	2 (2.1)*	1 (1.0)*
Deep sedation	18 (18.9)	16 (16.5)	51 (52.0)*^,^ **

Data are expressed as number (%)

*P < 0.01 vs. 500 ml/h

**P < 0.01 vs. 1000 ml/h

**Table 5:** Pain during injection and satisfaction

Variable	500 ml/h	1000 ml/h	2000 ml/h
Pain during injection	7 (7.4)	10 (10.3)	9 (9.2)
Patients’ VAS	9.5 ± 0.6	9.8 ± 0.4*	9.7 ± 0.5**
Endoscopist’s VAS	8.0 ± 0.8	9.3 ± 0.9*	9.4 ± 0.8*

Data are expressed as means ± SD or number (%)

*P < 0.01 vs. 500 ml/h

**P < 0.05 vs. 1000 ml/h

### Satisfaction

Mean VAS scores were significantly decreased in patients treated with propofol at 500 ml/h in comparison with those receiving the drug at 1000 ml/h (*P* < 0.01) and 2000 ml/h (*P* < 0.05). Average VAS scores for endoscopist’s satisfaction were significantly increased in the 1000 and 2000 ml/h groups compared with the 500 ml/h group (*P* < 0.01) (Table 5).

## Discussion

This study aimed to assess three infusion rates of propofol for their impact on sedation and adverse effects for outpatient upper gastrointestinal endoscopy. Results showed that the incidences of hypoxemia, hypotension, and deep sedation and the recovery times were significantly increased in patients treated at 2000 ml/h in comparison with the 500 and 1000 ml/h groups. Compared with the 500 ml/h group, motor activity of the extremities, cough, nausea, hiccough, and awareness were significantly decreased and the mean scores on the visual analogue scale for endoscopist’s and patients’ satisfaction were significantly increased in the 1000 and 2000 ml/h groups.

Here, BIS was used as a new way to assess deep sedation frequency. To improve the sensitivity and specificity, deep sedation was defined as BIS ≤65 [16–18, 20]. Based on this definition, deep sedation occurred in 18, 16 and 51 % of the patients in the 500, 1000 and 2000 ml/h groups, respectively. Although propofol was administered at rates targeting conscious sedation, deep sedation was often obtained at 2000 ml/h. However, it should be noted that BIS monitoring accuracy for detecting deep sedation is still controversial [17]. Differences in BIS values were observed between the groups at loss of consciousness, endoscope reaching the glottis, endoscope reaching the duodenum and the end of endoscopy. In addition, a greater decrease in BIS was observed after treatment at 2000 ml/h in comparison with the 500 and 1000 ml/h groups. Prior to these time points, BIS values were similar between the three groups. Therefore, it may be assumed that these differences might result from the propofol dose used for induction (2.9 mg kg^−1^) during procedure initiation. The BIS recordings are reflective of the previous 15 s of brain activity [18], and BIS values exhibit good correlation with consciousness [9]. Deep sedation associated adverse effects were readily alleviated with common clinical procedures. Although brain propofol amounts were not measured here, the delay from infusion completion and complete response suppression in the 2000 ml/h group might corroborate findings in sheep by Ludbrook et al. [21]. Our method eliminated spuriously high BIS scores that arise from patient stimulation during modified observer’s assessment of alertness/sedation (MOAA/S) scale evaluation. By frequently stimulating the patient for MOAA/S, the sedation target might require more medication and a higher MOAA/S score than if the MOAA/S score were assessed less frequently. The drug dose and sedation depth were similar when the two methods were used [22]. In the present study, the patients were evaluated, and the drugs were adjusted according to BIS rather than MOAA/S score. BIS is superior to MOAA/S in simplicity, offering more continuous measurements. It therefore objectively evaluate sedation in individuals submitted to endoscopy [16].

Low infusion rates in the 500 ml/h group did not abolish patient reflexes although eyelids remained closed, which probably led to the difference in endoscopy duration since less movements results in an easier procedure. Our findings indicate that increased propofol infusion rate is associated with decreased BIS values, in agreement with previous studies [20, 23]. Insufficient propofol administration results in patient excitement, and additional propofol must be given for endotracheal intubation. Therefore, there is a need for proper anesthetic amounts during endoscopy, prior to the procedure. Slow induction is more likely to cause excitatory side effects. In the present study, deeper anesthesia was achieved with faster injection, as indicated by the loss of the eyelash reflex.

Fanti et al. [24] have reported that the most common complications in gastrointestinal endoscopy were related to the sedation and not to the procedure itself. These complications include cardio-respiratory adverse events such as hypoxemia, hypoventilation, apnea, dysrhythmias, hypotension and vasovagal episodes [24]. As shown above, no differences in heart rates among the three groups were observed.

Hypoxemia incidence rates were 10, 15 and 40 % in the 500 ml/h, 1000 ml/h and 2000 ml/h groups, respectively, making it the most common complication. However, hypoxemia responded to oxygen supplementation. Relatively high transient mild hypoxemia rates in all groups might result from the close patient monitoring during sedation. Such data contribute to fueling the debate on the routine administration of supplemental oxygen during upper gastrointestinal endoscopy. The overall incidence of apnea was 22.4 % as shown above, in agreement with previous studies (20–30 %) [25, 26] assessing younger subjects with apnea defined as a ventilatory pause >40 s.

Mild hypotension is a frequent complication of propofol sedation. The reduction in hypotension frequency in the 500 and 1000 ml/h groups compared with the 2000 ml/h group is consistent with the results of previous studies [7–10, 12–14, 27]. This mild hypotension rarely has adverse clinical consequences and usually requires no intervention. The hypotensive episodes in the present study were transient and did not require pharmacological treatment.

Studies have demonstrated that a slower propofol injection rate decreases cardiovascular effects ([12–14, 28, 29]. However, slow injection rates might result in longer induction times [12–14, 30]. As shown above, increased induction times were observed with lower injection speeds, corroborating previous studies [12–14, 30, 31]. Here, lower infusion rates caused increased induction times, resulting in reduced induction doses of propofol, without differences in the total propofol dose at induction end. Propofol was administered at 2.4 mg/kg in average at 500 and 1000 ml/h, which is in the standard range of 2–2.5 mg/kg [32].

This study has some potential limitations. First, the patients were exclusively Chinese individuals, and some conclusions might not be globally generalized. Second, we did not evaluate the economics or the side effects of the regimens after the patients were discharged. Third, all patients received oxygen before induction, and the controversy about routine oxygen administration [33, 34] could not be addressed by the present study. Fourth, men and women were included, and it is well known that there are gender differences in anesthesia [35]. Fifth, different levels of sedation depth could be associated different rates of adverse effects, which could confound the results. Sixth, only mild/moderate sedation was studied, and the results may not apply to deep sedation. Seventh, no cost-effectiveness analysis was performed, but it may be assumes that optimizing the sedation process should lead to decrease costs. Eighth, some patients had a history of endoscopy, which could influence their experience of subsequent procedures. Finally, we did not follow-up the patients after discharge to monitor eventual longer-term adverse effects.

The present study suggests that a propofol infusion rate of 1000 ml/h appears to be the most appropriate rate for upper gastrointestinal endoscopy anesthesia, resulting in fewer adverse effects and improved satisfaction. Therefore, this rate should be used for upper gastrointestinal endoscopy anesthesia in selected patients corresponding to the inclusion criteria of this study. These results suggests that it is possible to optimize propofol administration in these patients. Nevertheless, further studies are necessary to broaden the generalisability of these results.

## Conclusion

In conclusion, a propofol infusion rate of 1000 ml/h appears to be the most appropriate rate for upper gastrointestinal endoscopy anesthesia. This rate resulted in fewer complications and adverse reactions during endoscopy, as well as in improved endoscopist satisfaction.

### Data availability

Data are available upon request to the corresponding author.

## Abbreviations

IV: intravenous
VAS: visual analogue scale
